# Glycyrrhizinate Monoammonium Cysteine-Loaded Lipid Nanoparticles Allow for Improved Acute Liver Injury Therapy

**DOI:** 10.3390/pharmaceutics17010090

**Published:** 2025-01-12

**Authors:** Yunjie Xu, Pinghui Li, Shiran Sun, Yulin Chen, Lixia Feng, Dawei Jiang, Chidan Wan, Jianbo Li, Xiong Cai

**Affiliations:** 1 Department of Hepatobiliary Surgery, Union Hospital, Tongji Medical College, Huazhong University of Science and Technology, Wuhan 430022, China; xuyunjie09wk@163.com (Y.X.);; 2 The School of Basic Medical Sciences, Inner Mongolia Medical University, Hohhot 010050, China; lph_edu@163.com; 3 Department of Nuclear Medicine, Union Hospital, Tongji Medical College, Huazhong University of Science and Technology, Wuhan 430022, China; 4 Department of Nuclear Medicine, The Affiliated Hospital of Inner Mongolia Medical University, Hohhot 010050, China

**Keywords:** acute liver injury, lipid nanoparticles, glycyrrhizinate monoammonium cysteine, FAPI, PET/CT

## Abstract

**Background:** Acute liver injury (ALI) is a prevalent and potentially lethal condition globally, where pharmacotherapy plays a vital role. However, challenges such as rapid drug excretion and insufficient concentration at hepatic lesions often impede the treatment’s effectiveness. **Methods:** We successfully prepared glycyrrhizinate monoammonium cysteine (GMC)-loaded lipid nanoparticles (LNPs) using high-pressure homogenization. The characterization and safety of the LNPs were measured using electrophoretic light scattering (ELS), transmission electron microscopy (TEM), dynamic light scattering (DLS), cytotoxicity assays, and hemolysis tests. The distribution of LNPs in mice was explored using fluorescence labeling methods. The encapsulation efficiency of LNP-GMC was detected using High-Performance Liquid Chromatography (HPLC), and its slow-release effect on GMC was assessed through dialysis. The therapeutic effects of LNP-GMC and pure GMC on the ALI model were evaluated using fibroblast activation protein inhibitor (FAPI) PET imaging, blood biochemical indicators, and liver pathology slices. **Results:** The encapsulation of GMC in LNPs enhances drug stability and prolongs its hepatic retention, significantly improving its bioavailability and sustained release within the liver. This study also explores the expression of fibroblast activation protein (FAP) in ALI, employing ^68^Ga-FAPI PET/CT imaging for effective differentiation and assessment of liver injury. **Conclusions:** Our results suggest that LNPs offer an enhanced therapeutic approach for ALI treatment, reducing the required drug dosage, and ^68^Ga-FAPI PET/CT imaging provides a novel method for diagnosis and treatment assessment. This study contributes valuable insights into the utilization of LNPs in liver disease treatment, presenting a promising direction for future clinical applications.

## 1. Introduction

Acute liver injury (ALI), a condition marked by rapid onset, extensive hepatocyte necrosis, and impaired liver function, is globally prevalent and potentially life-threatening [1]. ALI is frequently induced by factors such as viral hepatitis infection and excessive intake of hepatotoxic drugs or substances [2,3,4]. Without timely detection and management, ALI can progress to acute liver failure (ALF), significantly endangering life [5]. Treatment typically involves nonsurgical methods, including hepatoprotective and anti-inflammatory drugs, complemented by liver transplantation in severe cases [6]. Although liver transplantation is a fundamental curative approach [7], its high risks and costs often relegate it to a last-resort option. Consequently, the effectiveness of pharmacological interventions is critical in the management of ALI patients.

A key factor in the development of ALI is the overproduction of reactive oxygen species (ROS) and consequent oxidative stress [8,9]. Therefore, strategies to eliminate excessive ROS are crucial for improving the liver’s microenvironment and mitigating the disease’s progression [10]. Glycyrrhizinate monoammonium cysteine (GMC) [11] is a composite drug that includes two components, glycyrrhizic acid and cysteine. It is currently employed clinically as a medication for clearing excessive ROS. GMC exhibits various effects, such as antioxidant [12], anti-inflammatory [13,14], and antiviral [15] properties, making it one of the primary drugs used in nonsurgical treatments [16]. However, challenges such as GMC’s rapid excretion and limited accumulation in the liver diminish its bioavailability, sometimes hindering the achievement of therapeutic effects within safe dosage parameters [17,18].

Nanotechnology has garnered significant interest in recent years [19]. The utilization of chemically functionalized nanoparticles, such as quantum dots (QDots) [20] and LNPs [21], has revolutionized targeted drug delivery, particularly to the liver. This innovative approach significantly enhances the bioavailability of medications for ALI, improves the precision of drug targeting, and minimizes adverse drug reactions [22,23,24]. LNPs stand out due to their high encapsulation efficiency, structural stability, and straightforward preparation process [25], making them a focal point in drug formulation research [26].

Diagnosis of ALI predominantly relies on biochemical markers like alanine aminotransferase (ALT) and aspartate aminotransferase (AST) [27]. While imaging diagnostics provide valuable support, they often fall short in accurately delineating the extent and ongoing progression of liver damage, thus posing challenges in effectively evaluating the impact of nonsurgical treatments. Recent advancements include the development of fluorescent probes responsive to alterations in the liver’s internal milieu during ALI, such as neutrophil aggregation [28] and lysosomal viscosity changes [29]. Nonetheless, these techniques currently lack specificity, and their efficacy in the complex environment of the human body is yet to be fully established.

Concurrently, the inflammatory response in ALI triggers liver fibrogenesis pathways, primarily through the activation of fibroblasts [30]. Fibroblast activation protein (FAP), a cell surface glycoprotein belonging to the dipeptidyl peptidase-4 (DPP-4) enzyme family, is notably expressed on activated fibroblasts [31]. The fibroblast activation protein inhibitor (FAPI), a specific probe for FAP, has gained clinical approval predominantly for cancers like breast, colorectal, pancreatic, prostate, and lung cancer, all of which show pronounced connective tissue proliferation [32]. Research indicates the potential of FAPI in evaluating chronic liver injury [33]; however, its application in ALI scenarios remains unexplored.

In this study, we aim to deliver GMC to the liver via LNP loading for increased local drug retention and potentially hepatic bioavailability. Additionally, by targeting FAP and utilizing PET/CT imaging, this research assesses the effectiveness of drug-loaded treatments. We further investigate the potential of ^68^Ga-FAPI as a specific probe in the diagnosis and treatment monitoring of ALI.

## 2. Materials and Methods

### 2.1. Animals and Materials

A total of 20 *BALB/c* male mice (6–8 weeks, 17–20 g) were obtained from Beijing Vital River Laboratory Animal Technology Co., Ltd. (Beijing, China). The mice were housed in the SPF system at the Experimental Animal Center of Huazhong University of Science and Technology. The housing conditions were as follows: a light cycle from 8:00 to 20:00, an ambient temperature of 24 ± 2 °C, relative humidity ranging from 40% to 70%. The facility utilized an independent ventilation system with a room air exchange rate of at least 15 times per hour, and within the cages, ventilation occurred 30 to 100 times per hour. The mice were provided SPF-grade feed and purified water, with bedding changed 2 to 3 times per week. By cervical dislocation, the mice were euthanized, and any procedures that could potentially cause distress during the experimental process were preceded by isoflurane gas anesthesia for the mice.

Hydrogenated soybean phosphatidylcholine (HSPC), DSPE-mPEG2000, and cholest erol were sourced from Xi’an Ruixi Biological Technology Co., Ltd. (Xi’an, China). DiR was obtained from Umibio Science and Technology Group (Shanghai, China). The BCA Protein Assay Kit and Cell Counting Kit-8 were procured from Beyotime Biotechnology, China. FAPI-04 was acquired from Nanchang Tanzhen Biological Technology Co., Ltd. (Nanchang, China). All chemical reagents were purchased from Aladdin (Shanghai, China).

### 2.2. Preparation of LNP

A mixture of 95.8 mg HSPC, 30.0 mg DSPE-mPEG2000, and 31.9 mg cholesterol was dissolved in preheated anhydrous ethanol. Subsequently, 10 mL of ammonium sulfate solution (250 mM) was added to a round-bottom flask and preheated in a 65 °C water bath. This was followed by magnetic stirring to create a vortex, into which the organic phase was injected. The LNPs were formulated through a combination of ethanol injection and 200 nm polycarbonate film extrusion. Each component of the LNP formulation plays a distinct role. HSPC Provides structural stability and rigidity to the LNP bilayer. DSPE-mPEG2000 enhances particle sterility and stability by reducing aggregation and minimizing opsonization, thereby extending circulation time. Cholesterol stabilizes the LNP structure and modulates membrane fluidity.

### 2.3. Measurement of LNPs Characterization

The particle size of the LNPs was determined using dynamic light scattering (DLS) with a Malvern Zetasizer Nano ZSE (Malvern Instruments, Malvern, UK). The zeta potential was measured using the electrophoretic light scattering (ELS) technique on the same instrument. For morphology assessment, a transmission electron microscope (TEM, Joel, Tokyo, Japan) was used. To prepare and image LNPs using TEM, first, the LNPs are concentrated to 1 mg/mL. A TEM grid is cleaned and coated with a small drop of the LNP solution, followed by negative staining with a reagent like phosphotungstic acid (PTA) to enhance contrast. The excess stain is blotted away, and the sample is left to dry completely. Once dry, the grid is placed in the TEM for imaging.

### 2.4. Measurement of LNPs Stability 

An amount of 1 mg of LNPs was dissolved in 10 mL of PBS and incubated at 37 °C. Particle size was measured at intervals of 4, 12, 24, 48, and 96 h using DLS. Additionally, 1 mg of LNPs was incubated with 10 mL of 10% fetal bovine serum (FBS) at 37 °C, and particle size and dispersibility were measured at the same time points (4, 12, 24, 48, and 96 h) using DLS.

### 2.5. Cytotoxicity Analysis of LNPs In Vitro

LNP cytotoxicity was evaluated using the Cell Counting Kit-8 (CCK-8). MIHA cells, cultured in DMEM supplemented with 10% FBS, were maintained in a humidified incubator at 37 °C with 5% CO_2_. After seeding 5000 cells per well in a 96-well plate and overnight incubation, 10 μL of LNPs at specified concentrations was added to each well and further incubated for 24h at 37 °C. Subsequently, 10 μL of CCK-8 solution was added, followed by a 4h incubation at 37 °C. Absorbance values were measured at 450 nm using a Biotek Epoch microplate reader (BioTek Instruments, Winooski, VT, USA).

### 2.6. Hemolysis Assay

To assess LNP toxicity, cell hemolysis was tested at concentrations of 10, 50, and 100 µg at 37 °C for one hour. H_2_O and PBS served as positive and negative controls for hemolysis, respectively. After one hour, the reaction mixtures were centrifuged at 13,000× *g* for 10 min. Then, 100 µL of supernatant from each well was transferred to a new 96-well plate. Absorbance values at 540 nm were measured using a Biotek Epoch microplate reader (USA). Hemolysis percentage was calculated using the following formula:

% Hemolysis = ([A_540_ − minA_540_]/[maxA_540_ − minA_540_]) × 100%

where A_540_ is the average absorption at 540 nm, minA_540_ is the average absorption of PBS, and maxA_540_ is the average absorption of H_2_O.

### 2.7. Encapsulation Efficiency (EE%) of LNP

GMC was added to 100 μL of LNP, thoroughly mixed, and then heated in a 65 °C water bath for 30 min. PBS dialysis, using a dialysis membrane with a molecular weight cutoff of 100 kD, was performed to remove any unencapsulated GMC, resulting in the formation of LNP-GMC. High-Performance Liquid Chromatography (HPLC) was utilized to determine the drug encapsulation efficiency of the LNPs. LNP-GMC samples, both pre- and post-dialysis, were placed into EP tubes, mixed with acetonitrile at a 1:1 volume ratio, vortexed for 30 s, and then lysed. The analysis was conducted using a mobile phase of 90% acetonitrile at a flow rate of 1 mL/min, employing an Aquasil C18, 150 × 4.6 mm chromatography column (Thermo Fisher Scientific, Waltham, MA, USA). Absorbance was measured at 256 nm.

### 2.8. In Vitro Drug Release Study

For the drug release study, GMC (0.3 mg/100 µL) and LNP-GMC (0.3 mg/100 µL) were separately added into 100 KD dialysis bags, then placed in 5 mL of PBS (pH = 7.4) and incubated at 37 °C. At intervals of 4, 8, 12, 16, 20, and 24 h, 10 µL of the external solution was sampled, with an equal volume of PBS added after each sampling. The absorbance of GMC in the external solution was measured at 230 nm using a Biotek Epoch microplate reader (USA).

### 2.9. Preparation of DiR-LNP

DiR (0.1 mg), with an excitation wavelength of 645 nm, was dissolved in 50 µL of PBS. This solution was then added to 100 µL of LNPs at a concentration of 2 mg/mL DiR, thoroughly mixed, and incubated in a 65 °C water bath for 1 h. Dialysis was performed to remove free fluorescent dyes, resulting in the formation of DiR-LNP.

### 2.10. Pharmacokinetic Study

DiR and DiR-LNP were intravenously injected into BALB/c mice (8 weeks old, female, 18–20 g). Fluorescence imaging was conducted using a small animal in vivo optical imaging platform (PerkinElmer IVIS Lumina III, Waltham, MA, USA) at time points of 0.5 h, 1 h, 2 h, 4 h, 8 h, 16 h, 24 h, and 72 h post-injection. At the 72 h mark, the mice were euthanized, and their heart, liver, spleen, lung, and kidney were extracted for fluorescence imaging analysis. The intensity of fluorescence in these organs was quantified.

### 2.11. Construction of ALI Model

To construct the ALI model, carbon tetrachloride (CCl_4_) was diluted with corn oil in a 1:10 ratio, creating a 10% CCl_4_ solution. The mice were weighed, and the 10% CCl_4_ solution was administered at a dosage of 8 μL/g via gastric gavage.

### 2.12. GMC Drug Treatment

In this experiment, all drug administrations were performed via tail vein injection in mice. The dosage was based on the maximum safe therapeutic dose for humans, which is 3 μg/g, adjusted according to the mice’s body weight. The LNP-GMC treatment group and the free GMC treatment group received the same single injection dose of the GMC drug.

### 2.13. Animal Grouping

In this experiment, mice were divided into four groups: healthy mice, ALI mice, the free GMC drug-treated ALI group, and the LNP-GMC drug-treated ALI group. The healthy mice group did not receive any drug injections and were injected with an equal volume of PBS solution via the tail vein at the 2-h time point. The ALI group mice were modeled for ALI by intragastric administration of CCl_4_ and were injected with an equal volume of PBS solution via the tail vein 2 h post-administration. The free GMC drug-treated ALI group was modeled for ALI by intragastric administration of CCl_4_ and received free GMC drug injections via the tail vein at 2 h and 12 h post-administration. The LNP-GMC drug-treated ALI group was modeled for ALI by intragastric administration of CCl_4_ and received LNP-GMC drug injections via the tail vein 2 h post-administration. All groups underwent PET imaging 24 h after CCl_4_ administration, followed by blood sampling and major organ collection. The free GMC drug-treated ALI group underwent two blood sampling procedures, with samples taken at 12 h (named GMC_1_) and 24 h (named GMC_2_) post-administration.

### 2.14. Preparation of ^68^Ga-FAPI

^68^Ga was extracted using a 0.1 M hydrochloric acid solution. In a microtube, approximately 60 MBq of the ^68^Ga solution was combined with 0.28 mL of 0.25 M sodium acetate and 3 nmol of FAPI-04. The mixture was then incubated at 95 °C for 20 min. ^68^Ga-FAPI was purified using a C18 column (Waters Corporation, Milford, MA, USA).

### 2.15. Western Blot

Protein quantification was conducted using the BCA Protein Assay Kit. Proteins were mixed with 5× loading buffer and loaded onto a 10% SDS-PAGE. After blocking the membranes with 5% nonfat milk, they were incubated with primary antibodies overnight at 4 °C and then with horseradish peroxidase (HRP)-conjugated secondary antibodies for 1.5 h at room temperature. Protein levels were normalized relative to GAPDH.

### 2.16. PET/CT Imaging of ^68^Ga-FAPI

BALB/c mice were administered 3.7 MBq of ^68^Ga-FAPI through the tail vein. PET imaging was performed using a micro-PET/CT scanner (RAYCAN Technology Co, Ltd., Suzhou, China) at 15, 30, 60, and 120 min post-injection. PET data were reconstructed using the Ordered Subsets Expectation Maximization 3D or Maximum A Posteriori (OSEM3D/MAP) algorithm. The liver was then harvested and weighed, and its radioactivity was measured using a gamma counter (PerkinElmer WIZARD 2470-0010, Waltham, MA, USA). The percentage of injected dose per gram of tissue (%ID/g) was calculated accordingly.

### 2.17. Serum Levels of ALT and AST

Collected blood samples were placed in centrifuge tubes and allowed to clot naturally at room temperature for 10–20 min. The samples were then centrifuged at 2000–3000 rpm for 15 min, and the supernatant was collected. ALT and AST levels were determined using assay kits from Autobio, Zhengzhou, China.

### 2.18. Hematoxylin and Eosin Staining

Tissue samples were fixed in 4% paraformaldehyde, dehydrated in a graded series, embedded in paraffin, and sectioned into 4 µm slices. The slices were then stained with hematoxylin and eosin (H&E).

### 2.19. Statistical Analysis

Quantitative data were presented as mean ± standard deviation. Statistical significance was assessed using Student’s *t*-test, with a *p*-value of less than 0.05 considered statistically significant.

## 3. Results

### 3.1. Characterization of LNPs

**Figure 1:**
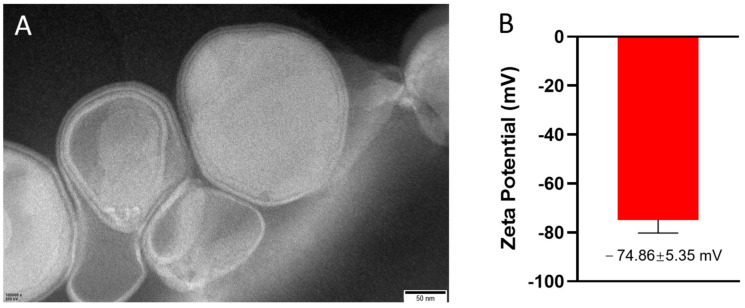
Characterization of LNP materials: (**A**) TEM image of LNPs; (**B**) zeta potential measurement of LNPs.

The particle size of LNPs is critical for their internal drug transport efficiency, since uniformed nanoparticle sizes typically demonstrate enhanced stability in biological fluids, a key factor for effective drug delivery [34]. Our LNPs exhibited relatively regular spherical shapes with a unified diameter of approximately 200 nm, as observed through TEM and illustrated in Figure 1A. Additionally, the zeta potential of the LNPs, measured using ELS, was determined to be −74.86 ± 5.35 mv, as shown in Figure 1B. Further confirmation of the particle size and uniformity was obtained through DLS measurements, as detailed in Supplementary Table S1, which further demonstrates that the synthesized liposomes have a size that aligns with our expectations and are relatively uniform. These characteristics suggest that our LNPs, with their consistent size around 200 nm, are well-suited for drug delivery to the liver. 

### 3.2. Stability of LNPs

For effective drug transport to the liver via the bloodstream, it is essential for the delivery materials to maintain strong stability to avoid premature rupture, which could impede targeted delivery. To enhance the stability of LNPs and reduce their binding with albumin and clearance by the immune system, we incorporated DSPE-mPEG2000 into the synthesis formula to achieve longer liver retention time and better sustained-release effects. To assess this, LNPs were incubated with both PBS solution and 10% FBS at 37 °C. Particle size measurements, taken using DLS at various time intervals (4, 12, 24, 48, and 96 h), showed sizes of 196.96 ± 1.93 nm, 195.95 ± 2.00 nm, 197.32 ± 5.34 nm, 193.66 ± 4.51 nm, and 193.65 ± 4.53 nm for PBS, and 215.60 ± 3.01 nm, 218.55 ± 4.24 nm, 219.09 ± 4.01 nm, 223.02 ± 6.32 nm, and 221.42 ± 2.89 nm for FBS. The corresponding polydispersity indices were 0.08 ± 0.02, 0.09 ± 0.05, 0.09 ± 0.02, 0.10 ± 0.06, and 0.12 ± 0.03 for PBS, and 0.13 ± 0.02, 0.12 ± 0.03, 0.12 ± 0.03, 0.14 ± 0.03, and 0.10 ± 0.01 for FBS, as detailed in Supplementary Tables S1 and S2. These results indicate that the LNPs possess a stable structure, enabling them to circulate within the body without premature rupture and effectively transport the drug to the liver.

### 3.3. Biosafety of LNPs

**Figure 2:**
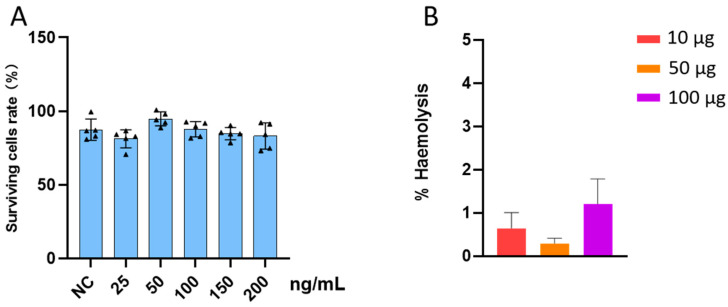
Safety experiments of LNP: (**A**) CCK-8 assay of LNP: cell viability at different concentrations of LNP; (**B**) hemolysis assay of LNPs (hemolysis fraction at different concentrations).

Ensuring the safety of materials used for internal drug transport is crucial. The cytotoxicity of LNPs was evaluated using the CCK-8, with the experiment spanning six groups at varying LNP concentrations: 0, 25, 50, 100, 150, and 200 ng/mL, as depicted in Figure 2A. Relative to the control group, no significant difference in cell survival rates was observed across these concentrations, suggesting negligible cytotoxicity. Moreover, to confirm the biosafety of LNPs in the bloodstream, a hemolysis experiment was conducted. The hemolysis rates for LNPs at 10, 50, and 100 µg, as shown in Figure 2B, were 0.64 ± 0.36%, 0.30 ± 0.12%, and 1.21 ± 0.58%, respectively, all below the 5% threshold [35], indicating that LNPs do not cause red blood cell destruction. These results collectively affirm that LNPs are noncytotoxic and hemolysis-free, validating their safety for internal drug transport.

### 3.4. Pharmacokinetics Study of LNPs

**Figure 3:**
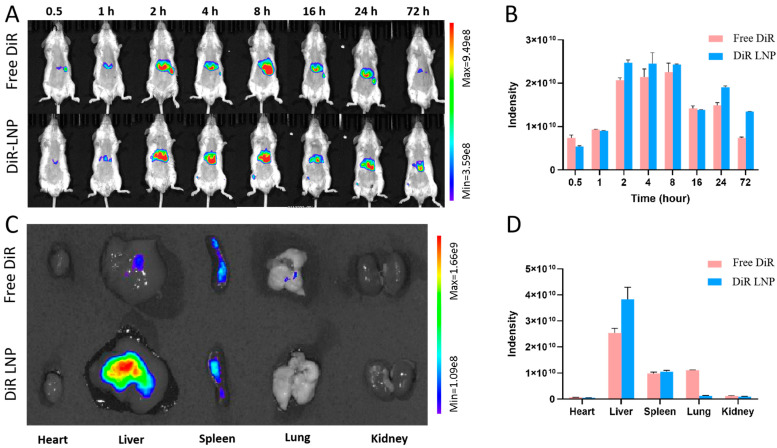
Fluorescent Imaging of DiR-LNP: (**A**) in vivo fluorescent imaging of DiR-LNP at different time points; (**B**) quantification of fluorescence intensity in the liver region from the images in (**A**); (**C**) fluorescent imaging of DiR-LNP in major organs at the 72 h time point; (**D**) quantification of fluorescence intensity in major organs from the images in (**C**).

Investigating the distribution of lipid bodies in mice is essential for understanding their efficacy as a drug delivery system. The internal distribution of these lipid bodies was examined using a fluorescence labeling technique. As illustrated in Figure 3A,B, the free fluorescent molecule DiR accumulated in the mouse liver, with its fluorescence signal significantly decreasing to approximately 0.75 × 10^10^ after 72 h. This decrease is attributed to the lipophilic nature of DiR [36], as lipophilic substances are more readily metabolized by the liver [37]. In contrast, DiR-LNP maintained a strong fluorescence signal intensity of approximately 1.3 × 10^10^ at 72 h, indicating prolonged retention of lipid bodies in the liver. Additionally, Figure 3C,D reveals that DiR-LNP accumulation in the liver was higher compared with free DiR, with lower fluorescence signals in other major organs, such as the heart, spleen, lungs, and kidneys. This study demonstrates that lipid bodies can effectively localize in the liver, laying a crucial foundation for their use in liver-targeted drug therapies.

### 3.5. Efficacy and Sustained Release of LNPs

To evaluate the efficacy and sustained release properties of LNP, we utilized a co-incubation method for encapsulating GMC within LNPs, followed by dialysis to eliminate any unencapsulated GMC. High-Performance Liquid Chromatography (HPLC) analysis revealed a significant change in the total peak area before and after dialysis (from 74,772.00 to 2857.00) (Supplementary Figure S1), resulting in an encapsulation efficiency of 44.71% for the LNP. The drug release profile of the purified LNP-GMC was further assessed using the dialysis method. The data indicated that free GMC passed through the dialysis membrane more readily than LNP-GMC, as evidenced by the faster increase in GMC concentration in the external solution. This difference implies that encapsulating GMC within LNPs leads to a sustained release effect, as depicted in Supplementary Figure S2.

### 3.6. Treatment Evaluation in ALI Model

**Figure 4:**
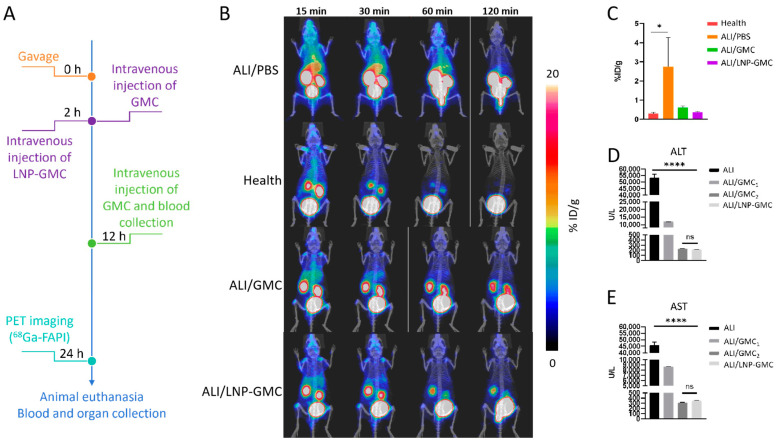
Treatment of ALI: (**A**) time schedule of ALI model establishment, treatment, and PET imaging using ^68^Ga-FAPI; (**B**) diagnostic evaluation of liver injury in different treatment groups using ^68^Ga-FAPI; (**C**) detection of liver uptake of ^68^Ga-FAPI using a gamma counter; (**D**) serum ALT levels in different treatment groups, *, **** indicates a significant difference with *p* < 0.01 and *p* < 0.0001, while ns indicates no significant difference; (**E**) serum AST levels in different treatment groups.

GMC, predominantly composed of amino acids and commonly used in the clinical treatment of ALI, typically requires intravenous infusion due to its short biological half-life. We encapsulated GMC in lipid bodies to leverage their accumulation and slow-release properties in the liver, aiming for an effective single-dose ALI treatment. As depicted in Figure 4A, an experiment was designed to compare the efficacy of lipid body-loaded GMC against regular GMC. The ALI model was divided into two groups (n = 3): one treated with a single dose of LNP-GMC and the other with two doses of standard GMC. The treatment effects were evaluated using ^68^Ga-FAPI PET imaging and blood biochemistry tests. Acute hepatitis causes hepatic stellate cells to differentiate into myofibroblasts expressing FAP on their surface [31], a finding confirmed through Western blot experiments (Supplementary Figure S3). This expression allowed the use of ^68^Ga-FAPI to diagnose and assess ALI. In Figure 4B, increased FAP expression due to liver damage was observed in the ALI model, evidenced by heightened radioactivity in the liver region. In healthy mice, ^68^Ga-FAPI was rapidly metabolized by the kidneys, showing minimal liver signal. This diagnostic approach was used to evaluate the therapeutic impacts of both GMC and LNP-GMC on ALI. Compared with the PBS-treated group, a significant decrease in liver radioactivity was noted, suggesting reduced liver injury. Notably, no significant difference in liver signal strength was observed between the GMC and LNP-GMC treatment groups, attributed to the slow release of GMC from lipid bodies. Gamma counter measurements (Figure 4C) showed significant differences in radioactive uptake between the PBS-treated and healthy groups (2.75 ± 1.52 %ID/g vs. 0.29 ± 0.07 %ID/g, *p* < 0.05). Compared with the PBS group, GMC treatment resulted in a significant decrease in the %ID/g value of liver ^68^Ga-FAPI, indicating a reduction in the degree of liver damage, but no substantial difference between the GMC and LNP-GMC groups. These findings align with the ^68^Ga-FAPI PET imaging results, demonstrating the feasibility of using ^68^Ga-FAPI PET imaging as a means for evaluating ALI.

### 3.7. Blood Biochemical Marker Assessment

In addition to PET imaging, serum analyses were conducted to assess liver injury, using ALT and AST as biomarkers (Figure 4D,E) [38]. Normal mouse serum levels are 21.00–44.00 U/L for ALT and 45.00–91.00 U/L for AST [39]. The PBS-treated group showed markedly elevated levels of ALT (53,490.20 ± 2500.52 U/L) and AST (45,922.00 ± 2268.65 U/L). The GMC-treated groups, labeled ALI/GMC_1_ (12 h post-treatment) and ALI/GMC_2_ (24-h post-treatment), demonstrated significant reductions in ALT and AST levels. The ALI/GMC_1_ group recorded 12,018.40 ± 25.17 U/L for ALT and 8762.90 ± 16.44 U/L for AST, while the ALI/GMC_2_ group showed 228.10 ± 4.49 U/L for ALT and 312.00 ± 8.80 U/L for AST. The LNP-GMC group had similar ALT (219.20 ± 1.20 U/L) and AST (352.30 ± 4.75 U/L) levels to the ALI/GMC_2_ group, indicating no significant difference, likely due to the sustained release of GMC from the lipid bodies. This serum analysis reinforces the potential of lipid encapsulation in enhancing the efficacy of GMC treatment for ALI.

### 3.8. Liver Tissue Staining Assessment

**Figure 5:**
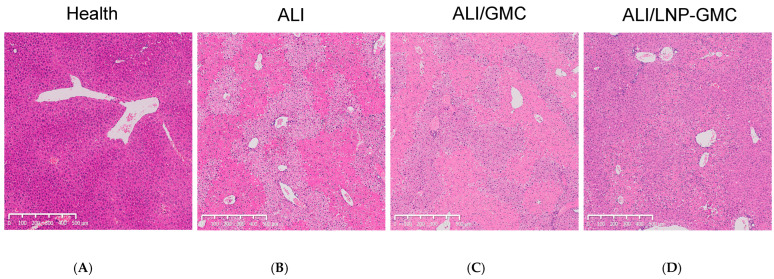
Liver H&E pathology: (**A**) pathology of normal mouse liver; (**B**) pathology of ALI mouse liver; (**C**) pathology of GMC-treated mouse liver; (**D**) pathology of LNP-GMC-treated mouse liver.

To substantiate our experimental findings, hematoxylin and eosin (H&E) pathological staining was performed on liver samples from various groups of mice, with the results presented in Figure 5. Figure 5A depicts the liver of a healthy mouse, exhibiting typical liver architecture and healthy hepatocytes. In contrast, Figure 5B reveals the liver pathology of an untreated ALI mouse, characterized by pronounced patchy hepatocyte necrosis. Meanwhile, Figure 5C displays the liver of a mouse treated with GMC, where patchy cell necrosis is still observable but is notably less severe compared with the untreated ALI group. Figure 5D illustrates the liver pathology of mice in the LNP-GMC group. In comparison with the GMC intervention group, the liver pathology of mice in the LNP-GMC group showed further improvement, with no apparent patchy necrosis observed. The cellular morphology and arrangement appeared relatively normal. This improvement is consistent with the findings from ^68^Ga-FAPI PET imaging, providing pathological validation for the effectiveness of our LNP-GMC drug-loaded treatment. It significantly ameliorates the necrotic conditions of liver cells in mice with ALI. 

## 4. Discussion

ALI is a serious global health issue with substantial mortality risk, and pharmacotherapy remains a cornerstone in its management. Despite advances in drug delivery and treatment options, challenges such as rapid drug excretion and limited concentration of therapeutic agents at hepatic lesion sites continue to restrict the efficacy of treatments. Furthermore, accurate and timely diagnosis and monitoring of ALI progression present ongoing obstacles. Our study aimed to address these challenges by investigating the efficacy of LNP-GMC in targeted hepatic delivery and evaluating the utility of ^68^Ga-FAPI PET/CT as a diagnostic tool. Through an extensive series of experiments, we assessed the effectiveness, safety, and biodistribution of LNP-GMC in ALI, and we discussed our findings in the context of similar studies.

Due to budgetary constraints related to PET/CT imaging costs, our study included a limited sample size for in vivo imaging, which restricts the statistical power of some of our findings. This limitation aligns with challenges encountered in similar studies, where the high cost of PET/CT often constrains sample sizes. Although our data provide initial evidence of ^68^Ga-FAPI PET/CT’s diagnostic potential for ALI, larger-scale studies are necessary to validate these findings and fully establish the clinical feasibility of FAPI PET/CT imaging in ALI diagnostics. Future studies with increased sample sizes could strengthen statistical power and provide more robust conclusions about ^68^Ga-FAPI’s diagnostic accuracy in varying degrees of liver injury.

In our study, during the synthesis of LNP, we used a 250 mM ammonium sulfate solution as the internal phase, creating a significant transmembrane ion concentration gradient. When mixing the LNPs with GMC for dialysis, we heated the solution to 65 °C. At this temperature, the lipid bilayer of the LNPs became more fluid, causing the lipid molecules to loosen their arrangement. This structural change allowed the GMC drug to penetrate into the LNPs’ core, leading to effective encapsulation of the GMC. Furthermore, during the synthesis of LNPs, we added DSPE-mPEG2000, which effectively prevents LNPs from binding to plasma proteins and being prematurely cleared by the mononuclear-phagocyte system, thereby extending the circulation time of LNPs [40]. LNPs can enter the liver through the pores of the liver sinusoidal endothelial cells. A portion of the LNPs may be phagocytosed by the hepatic reticuloendothelial system (RES) and delivered to lysosomes, where the drug is released after lysosomal dissolution. Another portion may bind to the low-density lipoprotein receptor (LDLR) and other lipid-associated receptors on the surface of liver cells and enter the cells via endocytosis to exert its therapeutic effect [21].

Our results demonstrate that LNP-GMC exhibited minimal cytotoxicity in vitro and did not induce significant hemolysis in vivo, suggesting that LNPs are a biologically safe medium for hepatic drug delivery. This finding aligns with previous studies supporting lipid-based carriers for hepatic applications, citing their biocompatibility and minimal adverse effects [41]. However, some studies have noted that interactions between nanoparticles and immune cells may induce subtle immune responses, even when biocompatibility is high [42]. Although we observed no significant immune responses in our experimental model, future research could further assess potential immune interactions under a range of clinical conditions to ensure that LNP-GMC is suitable for broad application in ALI patients.

LNPs of different sizes have different biological distribution and physical properties in vivo [43]. In the LNP synthesis process, we can control the particle size by optimizing factors such as lipid composition, ethanol-to-aqueous phase ratio, injection rate, and stirring speed [40]. In our study, to ensure both uniformity and a consistent diameter of approximately 200 nm, the synthesized mixture was subjected to physical extrusion through a 200 nm polycarbonate film. This process ensures the LNPs maintain their desired size while achieving a homogeneous distribution. Our study also showed that LNP-GMC significantly enhances the retention and sustained release of GMC in hepatic tissue. With controlled particle sizes around 200 nm, LNP-GMC demonstrated high stability and encapsulation efficiency, qualities that are crucial for liver-specific drug delivery. Nonetheless, it has been documented that interactions with serum proteins can sometimes alter nanoparticle stability, which could impact biodistribution and therapeutic efficacy [44]. In our experiments, we validated the stability of LNPs by co-incubating them with 10% FBS and observed the distribution of LNPs in vivo using DiR labeling. The LNPs were not significantly affected by serum proteins, which may be related to the addition of DSPE-mPEG2000 during LNP synthesis. However, considering the differences between humans and animals, we hope to obtain more convincing evidence, ideally in human studies, in the future.

Mechanistically, our study primarily focused on evaluating the therapeutic efficacy of LNP-GMC and validating ^68^Ga-FAPI PET/CT imaging rather than probing the molecular mechanisms underlying GMC’s effects on ALI. Although we observed increased FAP expression in the liver tissue of ALI mice, indicating its potential utility as a biomarker, a more in-depth investigation into FAP’s role in the pathogenesis of ALI is warranted. Understanding how FAP expression and fibroblast activation contribute to liver injury and repair could provide further insights into the therapeutic potential of targeting FAP in ALI. Additional studies could explore the molecular pathways involving FAP in liver damage and evaluate how ^68^Ga-FAPI PET imaging could reflect these pathways in real time.

Furthermore, ^68^Ga-FAPI PET/CT allowed us to effectively distinguish between healthy and damaged liver tissue, validating its potential for ALI diagnosis and therapeutic monitoring. Prior research has demonstrated that FAPI-based imaging effectively detects fibrotic changes in several organs [32]; however, due to the limitation of sample size and the lack of human data, the specific efficacy of ALI clinical diagnosis still cannot be accurately concluded.

In our therapeutic evaluation, LNP-GMC demonstrated superior efficacy compared with an equivalent dose of unencapsulated GMC, as evidenced by greater reductions in ALT and AST levels, further improved histopathological findings, and enhanced results on PET imaging. This finding is consistent with studies indicating that LNP encapsulation can enhance drug retention and stability, ultimately improving therapeutic outcomes [45]. However, manufacturing consistency remains an important consideration, as minor variations in LNP production can impact particle size, drug loading, and release characteristics [46]. To ensure consistent clinical outcomes, future research should focus on optimizing LNP production techniques and standardizing parameters to enhance reproducibility and quality control. At the same time, changing the structure of LNPs to achieve stronger targeting is also an important direction of future research.

In summary, our study demonstrates that LNP-GMC holds promise as a targeted drug delivery system for ALI treatment, addressing critical challenges in drug retention, safety, and diagnostic precision. However, additional studies with larger sample sizes, an expanded focus on immune interactions, and a deeper exploration of FAP’s molecular role in liver injury are essential for advancing LNP-based approaches and FAPI PET/CT imaging in ALI management. These findings contribute valuable insights to the growing fields of nanomedicine and molecular imaging in hepatology, supporting the development of safer and more effective liver disease treatments.

## 5. Conclusions

To conclude, our study underscores the dual benefits of utilizing LNPs (200 nm) as carriers for GMC—not only augmenting therapeutic efficacy but also minimizing the frequency of administration. Due to the sustained release effect of liposomes, a single dose is equivalent to two doses. Furthermore, the incorporation of ^68^Ga-FAPI as an imaging tool in ALI introduces a novel dimension for diagnosing and evaluating the treatment outcomes of acute liver injury. These discoveries unveil innovative strategies and avenues for the prospective pharmacological management of liver diseases, promising a substantial impact on clinical practices in the future.

## Data Availability

Upon reasonable request, data can be obtained from the corresponding author.

## Supplementary Materials

The following supporting information can be downloaded at https://www.mdpi.com/article/10.3390/pharmaceutics17010090/s1, Figure S1: HPLC detection of the encapsulation efficiency of LNP; Figure S2: The drug release of LNPs was confirmed through dialysis; Figure S3: Western blot experiment of FAP protein in the liver of ALI and normal mice; Tables S1 and S2: Material stability measurement of LNPs. DLS measurements of average particle size and polydispersity index of LNPs at different time points in PBS and 10% FBS.
