# Supplementary material for: Glycyrrhizinate Monoammonium Cysteine-Loaded Lipid Nanoparticles Allow for Improved Acute Liver Injury Therapy
Source: Pharmaceutics. 2025 Jan 12;17(1):90. doi: 10.3390/pharmaceutics17010090 (PMC11769283; doi:10.3390/pharmaceutics17010090)

Supplementary Table S1. DLS measurements of average particle size and polydispersity index of LNP at different time points in PBS.

Time (h)	Eff. Diam. (nm)	Polydispersity
4	196.96±1.93	0.08±0.02
12	195.95±2.00	0.09±0.05
24	197.32±5.34	0.09±0.02
48	193.66±4.51	0.10±0.06
96	193.65±4.53	0.12±0.03

Supplementary Table S2. DLS measurements of average particle size and polydispersity index of LNP at different time points in 10%FBS.

Time (h)	Eff. Diam. (nm)	Polydispersity
4	215.60±3.01	0.13±0.02
12	218.55±4.24	0.12±0.03
24	219.09±4.01	0.14±0.03
48	223.02±6.32	0.14±0.03
96	221.42±2.89	0.10±0.01

Supplementary Figure S1. HPLC detection of the encapsulation efficiency of LNP.

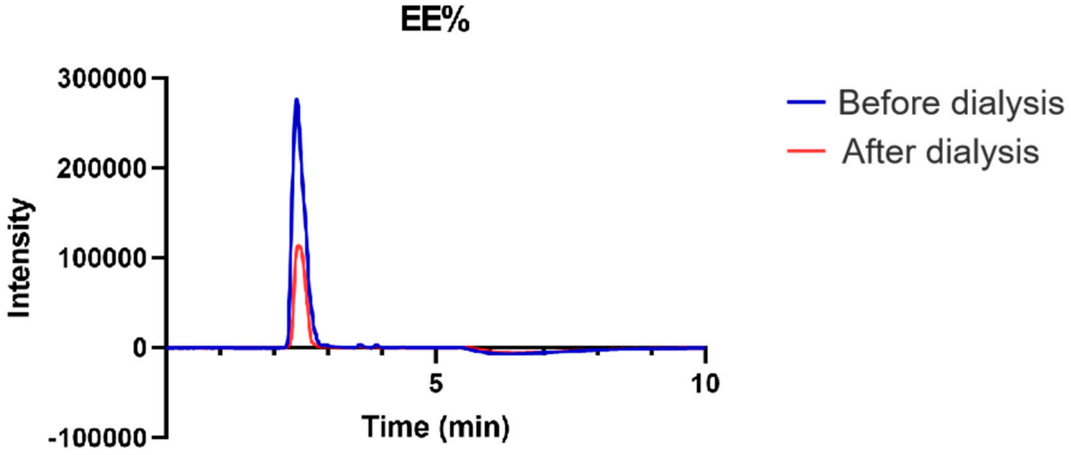

Supplementary Figure S2. The drug release of LNP was confirmed through dialysis.

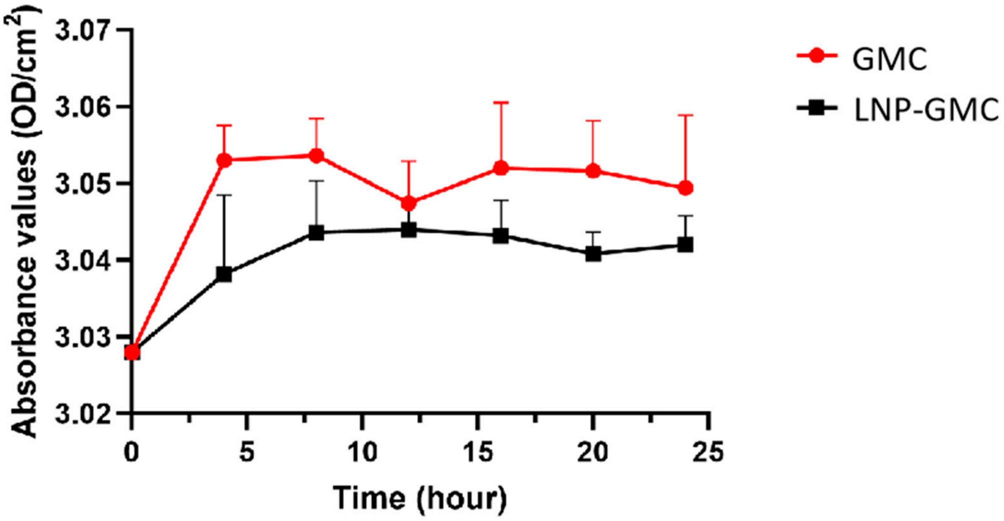

Supplementary Figure S3. Western blot experiment of FAP protein in the liver of ALI and normal mice.

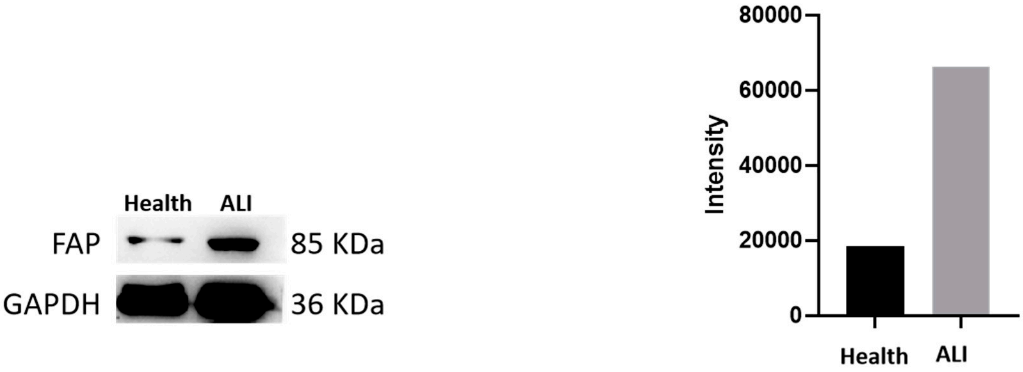

Supplement: Supplementary file 1 [file pharmaceutics-17-00090-s001.zip › pharmaceutics-3391746-supplementary.pdf]
